# Integrating gene set analysis and nonlinear predictive modeling of disease phenotypes using a Bayesian multitask formulation

**DOI:** 10.1186/s12859-016-1311-3

**Published:** 2016-12-13

**Authors:** Mehmet Gönen

**Affiliations:** Department of Industrial Engineering, Koç University, İstanbul, 34450 Turkey

**Keywords:** Gene set analysis, Nonlinear predictive modeling, Disease phenotypes, Multiple kernel learning, Cancer, Tuberculosis

## Abstract

**Background:**

Identifying molecular signatures of disease phenotypes is studied using two mainstream approaches: (i) Predictive modeling methods such as linear classification and regression algorithms are used to find signatures predictive of phenotypes from genomic data, which may not be robust due to limited sample size or highly correlated nature of genomic data. (ii) Gene set analysis methods are used to find gene sets on which phenotypes are linearly dependent by bringing prior biological knowledge into the analysis, which may not capture more complex nonlinear dependencies. Thus, formulating an integrated model of gene set analysis and nonlinear predictive modeling is of great practical importance.

**Results:**

In this study, we propose a Bayesian binary classification framework to integrate gene set analysis and nonlinear predictive modeling. We then generalize this formulation to multitask learning setting to model multiple related datasets conjointly. Our main novelty is the probabilistic nonlinear formulation that enables us to robustly capture nonlinear dependencies between genomic data and phenotype even with small sample sizes. We demonstrate the performance of our algorithms using repeated random subsampling validation experiments on two cancer and two tuberculosis datasets by predicting important disease phenotypes from genome-wide gene expression data.

**Conclusions:**

We are able to obtain comparable or even better predictive performance than a baseline Bayesian nonlinear algorithm and to identify sparse sets of relevant genes and gene sets on all datasets. We also show that our multitask learning formulation enables us to further improve the generalization performance and to better understand biological processes behind disease phenotypes.

## Background

Predictive modeling is frequently used to find molecular signatures of disease phenotypes from genomic data, which helps us better understand underlying biological processes behind phenotypes and reduce data acquisition cost for future clinical samples by doing targeted profiling instead of genome-wide screens. To this aim, supervised machine learning methods such as linear classification and regression algorithms are trained to predict disease phenotypes, and features with relatively higher importance values (e.g. features with larger magnitude weights) in these parametric models are included into the signature. However, as illustrated by existing studies [1, 2], molecular signatures identified by such algorithms may not be robust due to small sample size or highly correlated nature of genomic data.

Gene set analysis methods try to identify gene sets on which disease phenotypes are dependent by calculating an enrichment score for each gene and transforming these scores into gene set scores using a summarization procedure [3]. The main advantage of these approaches is the ability to bring prior biological knowledge into the analysis in the form of biological pathways or sets of genes with similar biological functions [4], leading to more robust and clinically interpretable results than predictive modeling approaches. However, they usually assume linear dependencies between genomic data and phenotype, which may not reflect the underlying biology of disease, and have difficulties in using very small or large gene sets in the analysis.

To benefit from the best of both worlds, integrating gene set analysis and predictive modeling is already considered in many existing studies [5–7], which modify linear classification and regression algorithms to include gene set information while doing feature selection for molecular signature extraction. Even though this family of methods capture dependencies between genes, they still fail to capture nonlinear dependencies between genomic data and phenotype.

We suggest to integrate these two components using a nonlinear framework by extending our earlier Bayesian formulation [8]. Here, we develop a novel Bayesian multiple kernel learning algorithm, which trains a binary classifier with a sparse set of active gene sets using a sparsity-inducing prior, i.e. the spike and slab prior [9]. Using gene sets within a probabilistic formulation helps us identify more robust signatures even with small sample sizes. Using a kernel-based formulation enables us to capture nonlinear dependencies between genomic data and phenotype, and to use overlapping gene sets and gene sets with different sizes without any major concern. We also generalize our proposed formulation to multitask learning setting to model multiple related datasets (e.g. different patient cohorts profiled against the same phenotype) conjointly, leading to better predictive performance and more robust molecular signatures. To the best of our knowledge, [10] provides the first joint formulation of gene set analysis and nonlinear predictive modeling, which performs a survival analysis on breast cancer patients using both clinical and genomic data, using an existing discriminative multiple kernel learning algorithm. However, our approach has important advantages over their method: (i) more robustness on clinical datasets with small sample size due to its probabilistic nature, (ii) its ability to perform automatic model selection (e.g. determining the sparsity level of kernel weights) due to its fully Bayesian inference mechanism and (iii) its ability to model multiple related datasets conjointly due to its multitask learning variant.

We perform repeated random subsampling validation experiments on two cancer and two tuberculosis datasets to demonstrate the better predictive performance of our two algorithms over a baseline Bayesian nonlinear algorithm and to show the biological relevance of the genes and gene sets selected to disease phenotypes modeled.

## Materials

In this study, we use two cancer and two tuberculosis datasets, where we solve binary classification problems to predict phenotype values from genomic data and to extract molecular signatures of disease phenotypes.

### Diagnosis of micro-satellite instability in colorectal and endometrial carcinomas

Micro-satellite instability is a hypermutable phenotype caused by the loss of DNA mismatch repair activity. It is frequently observed in several tumor types such as colorectal, endometrial, gastric, ovarian and sebaceous carcinomas [11]. Tumors with micro-satellite instability do not respond to chemotherapeutic strategies developed for micro-satellite stable tumors, leading to its clinical importance. That is why we address the problem of predicting micro-satellite instability status of cancer patients from their gene expression data. We use two publicly available datasets provided by ‘the Cancer Genome Atlas’ (TCGA) consortium: (i) ‘colon and rectum adenocarcinoma’ (COADREAD) patients [12] and (ii) ‘uterine corpus endometrial carcinoma’ (UCEC) patients [13].

The phenotype values of cancer patients for both datasets are downloaded from the TCGA website (https://tcga-data.nci.nih.gov), which groups the patients into three categories: (i) ‘micro-satellite instability high’ (MSI-H), (ii) ‘micro-satellite instability low’ (MSI-L) and (iii) ‘micro-satellite stable’ (MSS). The preprocessed genomic characterizations of primary tumors from the patients (i.e. mRNA gene expression) are downloaded from https://www.synapse.org/%23%21Synapse:syn300013, where 20,530 normalized gene expression intensities are provided for each profiled primary tumor. We remove the patients with missing phenotype value or genomic data from further analysis. At the end, there are 261 and 330 patients with available phenotype value and genomic data for COADREAD and UCEC datasets, respectively. Table 1 summarizes the final datasets by listing the numbers of patients in each category together with the total number of patients.

**Table 1:** Summary of two cancer datasets

	Number of patients			
Dataset	MSI-H	MSI-L	MSS	Total
COADREAD	37	43	181	261
UCEC	108	27	195	330

*MSI-H* Micro-satellite instability high, *MSI-L* Micro-satellite instability low, *MSS* Micro-satellite stable

### Diagnosis of tuberculosis in adult and pediatric individuals

Tuberculosis is responsible for 1.5 million deaths in 2013 according to the World Health Organization, which makes it the second greatest killer due to a single infectious agent after HIV. It is also the leading cause of death for HIV-infected people. Its diagnosis is currently based on clinical and radiological features, sputum microscopy and tuberculin skin testing, which usually give false results in HIV-infected individuals [14]. New clinical diagnostic tests, especially for resource poor settings such as low-income countries with high rates of HIV, are needed to identify tuberculosis cases correctly for proper treatment. That is why we address the problem of predicting tuberculosis status of individuals from genome-wide RNA expression in host blood. We use two publicly available datasets of HIV-infected and -uninfected individuals from South Africa and Malawi: (i) adult individuals (ADULT) [14] and (ii) pediatric individuals (PEDIATRIC) [15].

The phenotype values and the genomic data for ADULT and PEDIATRIC datasets are downloaded from NCBI’s Gene Expression Omnibus using GEO Series accession numbers GSE37250 and GSE39940, respectively, where the individuals are grouped into three categories: (i) ‘active tuberculosis’ (ATB), (ii) ‘latent tuberculosis infection’ (LTBI) and (iii) ‘other disease’ (OD). These repositories contain background subtracted and quantile normalized intensities of 47 323 probes for each individual. There are 537 and 334 individuals with available phenotype and genomic data for ADULT and PEDIATRIC datasets, respectively. Table 2 summarizes the datasets by listing the numbers of individuals in each category together with the total number of individuals.

**Table 2:** Summary of two tuberculosis datasets

	Number of individuals			
Dataset	ATB	LTBI	OD	Total
ADULT	195	167	175	537
PEDIATRIC	111	54	169	334

*ATB* Active tuberculosis, *LTBI* Latent tuberculosis infection, *OD* Other disease

## Methods

We consider the problem of predicting phenotype values from genomic data using classification algorithms. Instead of training classifiers that use all available features, we want to develop classifiers that use very few but biologically relevant input features to identify a molecular signature of the phenotype and to reduce the data acquisition cost for test samples. However, the molecular signatures identified from, for example, gene expression data are not robust when we have limited training data [1, 2]. In such cases, we obtain different molecular signatures from different subsets of the same training set due to highly correlated nature of data, which makes knowledge extraction quite difficult. Instead, we can use our prior biological knowledge to group the input features and pick the relevant groups that are predictive of the phenotype while training the classification algorithm. We first discuss our proposed method that can learn a classifier and identify predictive gene sets simultaneously on a single dataset. We then explain how we extend our method to model multiple related datasets by identifying a common set of predictive gene sets across them.

### Sparse Bayesian multiple kernel learning

We formulate the prediction task as a binary classification problem defined on the genomic data, denoted as domain $\mathcal {X}$, and the phenotype, denoted as domain $\mathcal {Y}$. We are given an independent and identically distributed sample $\{\boldsymbol {x}_{i} \in \mathcal {X}\}_{i = 1}^{N}$ and a class label vector $\boldsymbol {y} = \{y_{i} \in \mathcal {Y}\}_{i = 1}^{N}$, where *N* is the number of data points, and $\mathcal {Y} = \{-1, +1\}$. We are also given a list of gene sets $\{\mathcal {I}_{m}\}_{m = 1}^{P}$, which encode our prior biological knowledge in terms of gene names, where $\mathcal {I}_{m}$ list the names of genes in the gene set *m*, which may be a set of genes from a biological pathway or a set of genes with similar biological functions, and *P* is the number of gene sets.

**Fig. 1:**
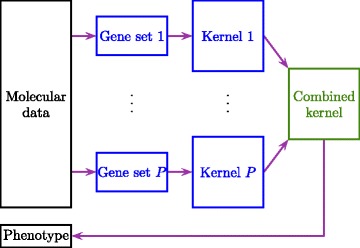
Schematic description of sparse Bayesian multiple kernel learning. For each gene set, the corresponding kernel considers only the features extracted from or related to the genes in this gene set. We then learn a weighted sparse combination of these kernels while training a binary classifier to predict the phenotype values

We choose to develop a nonlinear classifier to predict phenotype from genomic data using a kernel-based formulation due to its three main advantages [16]: (i) We can learn robust classifiers for tasks with very high dimensional representations such as genomic data and small sample size (i.e. large *p*, small *n*). (ii) We can learn better classifiers using nonlinear kernels such as the Gaussian kernel (i.e. kernel trick). (iii) We can use domain-specific kernels (e.g. graph and tree kernels for structured objects) to better capture the underlying biological processes [17]. To calculate similarities between the data points, we have multiple kernel functions defined over gene sets, namely, $\{k_{m}\colon \mathcal {X} \times \mathcal {X} \to \mathbb {R}\}_{m = 1}^{P}$, which are used to calculate the kernel matrices $\{\mathbf {K}_{m}\}_{m = 1}^{P}$. For each gene set, the corresponding kernel $k_{m}(\boldsymbol {x}_{i}, \boldsymbol {x}_{j} | \mathcal {I}_{m})$ considers only the features extracted from or related to the genes in $\mathcal {I}_{m}$. We choose to learn a weighted combination of the input kernels $\{\mathbf {K}_{m}\}_{m = 1}^{P}$ while training a binary classifier, which is known as multiple kernel learning [18], by extending our earlier Bayesian formulation [8] with a sparsity-inducing prior on the kernel weights. Figure 1 gives a schematic description of the proposed model.

#### Probabilistic model

Our proposed probabilistic model, called ‘sparse Bayesian multiple kernel learning’ (SBMKL), has three main parts: (i) finding kernel-specific latent variables using the same set of sample weights over the input kernels, (ii) assigning sparse weights to these latent variables using the spike and slab prior [9] and (iii) generating predicted outputs using the latent variables and these sparse weights together with a bias parameter.

The first part has the following distributional assumptions:

$$\begin{aligned} \lambda_{i} &\sim \text{Gamma}(\lambda_{i}; \alpha_{\lambda}, \beta_{\lambda}) &&\;\;\;\;\;\; \forall i \\ a_{i} | \lambda_{i} &\sim \text{Normal}\left(a_{i}; 0, \lambda_{i}^{-1}\right) &&\;\;\;\;\;\; \forall i \\ {g_{i}^{m}} | \boldsymbol{a}, \boldsymbol{k}_{m,i} &\sim \text{Normal}\left({g_{i}^{m}}; \boldsymbol{a}^{\top} \boldsymbol{k}_{m,i}, {\sigma_{g}^{2}}\right) &&\;\;\;\;\;\; \forall (m, i), \end{aligned} $$

where the superscript indexes the rows, the subscript indexes the columns, Normal(·;***μ***,**Σ**) represents the normal distribution with the mean vector ***μ*** and the covariance matrix **Σ**, and Gamma(·;*α*,*β*) denotes the gamma distribution with the shape parameter *α* and the scale parameter *β*. We generate the latent variables ***g*** ^*m*^ for each input kernel **K**_*m*_ using the same set of sample weights ***a***. Note that we need to use a small noise parameter *σ*_*g*_ while generating the latent variables to better generalize to test data points.

The second part has the following distributional assumptions:

$$\begin{aligned} \kappa &\sim \text{Beta}(\kappa; \zeta_{\kappa}, \eta_{\kappa}) && \\ s_{m} | \kappa &\sim \text{Bernoulli}(s_{m}; \kappa) &&\;\;\;\;\;\; \forall m \\ \omega &\sim \text{Gamma}(\omega; \alpha_{\omega}, \beta_{\omega}) && \\ e_{m} | \omega &\sim \text{Normal}\left(e_{m}; 0, \omega^{-1}\right) &&\;\;\;\;\;\; \forall m, \end{aligned} $$

where Beta(·;*ζ*,*η*) denotes the beta distribution with the shape parameters *ζ* and *η*, and Bernoulli(·;*π*) represents the Bernoulli distribution with the success probability parameter *π*. We generate a binary indicator variable *s*_*m*_ and a normally distributed weight *e*_*m*_ for each input kernel. The product of these two variables *s*_*m*_ *e*_*m*_ is a simple parameterization of the spike and slab prior, which is more amenable to approximate inference.

The third part has the following distributional assumptions:

$$\begin{aligned} \gamma &\sim \text{Gamma}(\gamma; \alpha_{\gamma}, \beta_{\gamma}) && \\ b | \gamma &\sim \text{Normal}(b; 0, \gamma^{-1}) && \\ f_{i} | b, \boldsymbol{e}, \boldsymbol{s}, \boldsymbol{g}_{i} &\sim \text{Normal}\left(f_{i}; (\boldsymbol{s} \circ \boldsymbol{e})^{\top} \boldsymbol{g}_{i} + b, 1\right) &&\;\;\;\;\;\; \forall i \\ y_{i} | f_{i} &\sim \text{Kronecker}(f_{i} y_{i} > \nu) &&\;\;\;\;\;\; \forall i, \end{aligned} $$

where ∘ represents the Hadamard product, and Kronecker(·) denotes the Kronecker delta function that returns 1 if its argument is true and 0 otherwise. The predicted outputs ***f***, similar to the discriminant outputs in support vector machines, are introduced to make the inference procedures efficient [19]. The nonnegative margin parameter *ν* is introduced to resolve the scaling ambiguity and to place a low-density region between two classes, similar to the margin idea in support vector machines, which is generally used for semi-supervised learning [20].

**Fig. 2:**
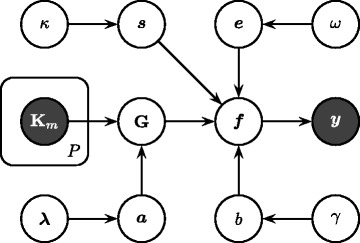
Graphical model of sparse Bayesian multiple kernel learning. Random variables are shown as *empty circles*, whereas observed variables are shown as *filled circles*. Hyper-parameters are ignored for simplicity

Figure 2 illustrates the proposed probabilistic model for binary classification with a graphical model.

#### Inference using variational Bayes

We need to infer the posterior distribution over the model parameters and the latent variables, which we denote as **Θ**={***λ***,***a***,**G**,*κ*,***s***,*ω*,***e***,*γ*,*b*,***f***}, given the input kernel matrices $\{\mathbf {K}_{m}\}_{m = 1}^{P}$ and the class labels ***y*** to find the predictive distribution for test data points. Unfortunately, exact inference for our proposed probabilistic model is intractable. Instead of using a computationally expensive Gibbs sampling approach [21], we choose to perform variational inference, which maximizes a lower bound on the marginal likelihood using an ensemble of factored posteriors to infer the joint parameter distribution [22].

We approximate the posterior distribution over the model parameters and the latent variables by a variational distribution:

$$\begin{array}{*{20}l} p(\mathbf{\Theta} | \{\mathbf{K}_{m}\}_{m = 1}^{P}, \boldsymbol{y}) &\approx q(\mathbf{\Theta}), \end{array} $$

where we assume that the variational distribution has a simpler form than the posterior distribution to make inference tractable. The inference problem can be defined as finding the nearest variational distribution to the posterior distribution with respect to a distance function. We perform mean-field variational Bayes, which measures the distance between distributions *q* and *p* using ‘the Kullback–Leibler divergence’ denoted as $\mathcal {KL}(q||p)$. We can decompose the log evidence as

$${{}\begin{aligned} \log p(\boldsymbol{y} | \{\mathbf{K}_{m}\}_{m = 1}^{P}) &= \underbrace{\int q(\mathbf{\Theta}) \log \frac{p\left(\mathbf{\Theta}, \boldsymbol{y} | \{\mathbf{K}_{m}\}_{m = 1}^{P}\right)}{q(\mathbf{\Theta})} \mathrm{d}\mathbf{\Theta}}_{{ \mathcal{L}(q)}} \\ & \quad +\! \underbrace{\int \!- q(\mathbf{\Theta}) \log \frac{p\left(\mathbf{\Theta} | \{\mathbf{K}_{m}\}_{m = 1}^{P}, \boldsymbol{y}\right)}{q(\mathbf{\Theta})} \mathrm{d}\mathbf{\Theta} }_{{\mathcal{KL}(q||p)}}, \end{aligned}} $$

where we assume without loss of generality that all model parameters and latent variables are continuous variables, and see that minimizing $\mathcal {KL}(q||p)$ amounts to maximizing the lower bound $\mathcal {L}(q)$.

We start by writing *q*(**Θ**) as a factorized approximation:

$${{}\begin{aligned} q(\mathbf{\Theta}) = q(\boldsymbol{\lambda}) q(\boldsymbol{a}) q(\mathbf{G}) q(\kappa) q(\boldsymbol{s}) q(\omega) q(\boldsymbol{e} | \boldsymbol{s}) q(\gamma) q(b) q(\boldsymbol{f}), \end{aligned}} $$

where we couple the weights ***e*** with the binary indicator variables ***s*** due to their strong correlation. Note that we choose not to have the factorization as *q*(***e***)*q*(***s***) because it gives a unimodal distribution, but the true posterior distribution may have exponentially many modes. To capture this multimodal structure, we choose to formulate the factorization as *q*(***e***|***s***)*q*(***s***), which can be approximated efficiently [23]. We then write $\mathcal {L}(q)$ in the form of expectations:

$${{}\begin{aligned} \mathcal{L}(q) &= \mathrm{E}_{q(\mathbf{\Theta})}[\log p(\mathbf{\Theta}, \boldsymbol{y} | \{\mathbf{K}_{m}\}_{m = 1}^{P})] - \mathrm{E}_{q(\mathbf{\Theta})}[\log q(\mathbf{\Theta})], \end{aligned}} $$

where we iteratively maximize $\mathcal {L}(q)$ with respect to each factor until convergence. The approximate posterior distribution of a specific factor *τ* can be found as

$$\begin{aligned} q(\tau) \propto \exp \left(\mathrm{E}_{q(\mathbf{\Theta} \backslash \tau)}\left[\log p\left(\mathbf{\Theta}, \boldsymbol{y} | \{\mathbf{K}_{m}\}_{m = 1}^{P}\right)\right]\right). \end{aligned} $$

#### Inference details

We define the factors for the first part of our probabilistic model as

$$\begin{aligned} q(\boldsymbol{\lambda}) &= \prod \limits_{i = 1}^{N} \text{Gamma}(\lambda_{i}; \alpha(\lambda_{i}), \beta(\lambda_{i})) \\ q(\boldsymbol{a}) &= \text{Normal}(\boldsymbol{a}; \mu(\boldsymbol{a}), \Sigma(\boldsymbol{a})) \\ q(\mathbf{G}) &= \prod \limits_{i = 1}^{N} \text{Normal}(\boldsymbol{g}_{i}; \mu(\boldsymbol{g}_{i}), \Sigma(\boldsymbol{g}_{i})), \end{aligned} $$

where *α*(·),*β*(·),*μ*(·), and *Σ*(·) denote the shape parameter, the scale parameter, the mean vector and the covariance matrix of their arguments, respectively. The approximate posterior distributions can be updated as

$$\begin{aligned} \alpha(\lambda_{i}) &= \alpha_{\lambda} + 1/2 \\ \beta(\lambda_{i}) & = \left(1 / \beta_{\lambda} + \langle {{a_{i}^{2}}} \rangle / 2\right)^{-1} \\ \Sigma(\boldsymbol{a}) &= \left(\text{diag}(\langle \boldsymbol{\lambda} \rangle) + \sigma_{g}^{-2} \sum \limits_{m = 1}^{P} \mathbf{K}_{m} \mathbf{K}_{m}^{\top}\right)^{-1} \\ \mu(\boldsymbol{a}) &= \Sigma(\boldsymbol{a}) \left(\sigma_{g}^{-2} \sum \limits_{m = 1}^{P} \mathbf{K}_{m} \langle (\boldsymbol{g}^{m})^{\top} \rangle\right) \\ \Sigma(\boldsymbol{g}_{i}) &= \left(\sigma_{g}^{-2} \mathbf{I} + \langle (\boldsymbol{s} \circ \boldsymbol{e}) (\boldsymbol{s} \circ \boldsymbol{e})^{\top} \rangle\right)^{-1} \\ \mu(\boldsymbol{g}_{i}) &= \Sigma(\boldsymbol{g}_{i}) \left(\sigma_{g}^{-2} [\boldsymbol{k}_{1,i} ~ \dots ~ \boldsymbol{k}_{P,i}]^{\top} \langle \boldsymbol{a} \rangle \right. \\ & \quad \left. + \langle f_{i} \rangle \langle \boldsymbol{s} \circ \boldsymbol{e} \rangle - \langle b \rangle\langle \boldsymbol{s} \circ \boldsymbol{e} \rangle\right), \end{aligned} $$

where 〈*h*(·)〉 denotes the posterior expectation as usual, i.e. E_*q*(·)_[*h*(·)].

The factors for the second part of our probabilistic model are defined as

$$\begin{aligned} q(\kappa) &= \text{Beta}(\kappa; \zeta(\kappa), \eta(\kappa)) \\ q(\boldsymbol{s}) &= \prod \limits_{m = 1}^{P} \text{Bernoulli}(s_{m}; \pi(s_{m})) \\ q(\omega) &= \text{Gamma}(\omega; \alpha(\omega), \beta(\omega)) \\ q(\boldsymbol{e} | \boldsymbol{s}) &= \prod \limits_{m = 1}^{P} \text{Normal}(e_{m} | s_{m}; \mu(e_{m} | s_{m}), \Sigma(e_{m} | s_{m})), \end{aligned} $$

where *ζ*(·),*η*(·) and *π*(·) denote the shape parameters and the success probability parameter of their arguments. We can update the approximate posterior distributions as

$${{\begin{aligned} \zeta(\kappa) &= \zeta_{\kappa} + \sum\limits_{m = 1}^{P} \langle s_{m} \rangle \\ \eta(\kappa) &= \eta_{\kappa} + P - \sum\limits_{m = 1}^{P} \langle s_{m} \rangle \\ \pi(s_{m}) &= 1 / (1 + \exp(-r_{m})) \\ \alpha(\omega) &= \alpha_{\omega} + P/2 \\ \beta(\omega) &= \left(1/\beta_{\omega} + \sum\limits_{m = 1}^{P} (\langle 1 - s_{m} \rangle \langle {e_{m}^{2}}|0 \rangle + \langle s_{m} \rangle \langle {e_{m}^{2}}|1 \rangle)/2\right)^{-1} \\ \Sigma(e_{m} | 0) &= 1 / \langle \omega \rangle\\ \mu(e_{m} | 0) &= 0 \\ \Sigma(e_{m} | 1) &= 1 / (\langle \omega \rangle + \langle \boldsymbol{g}^{m} (\boldsymbol{g}^{m})^{\top} \rangle) \\ \mu(e_{m} | 1) &= \Sigma(e_{m} | 1) \sum\limits_{i = 1}^{N} \left[(\langle f_{i} \rangle \,-\, \langle b \rangle) \langle {g_{i}^{m}} \rangle \,-\, \sum\limits_{l \neq m} \langle s_{l} \rangle \langle e_{l} | 1 \rangle \langle {g_{i}^{l}} {g_{i}^{m}} \rangle \right], \end{aligned}}} $$

where the auxiliary variable *r*_*m*_ is defined as

$${{}\begin{aligned} r_{m} &= \left\langle\log\frac{\kappa}{1 - \kappa}\right\rangle - \frac{1}{2} \langle {e_{m}^{2}} | 1 \rangle \langle \boldsymbol{g}^{m} (\boldsymbol{g}^{m})^{\top} \rangle \\ & \quad +\langle e_{m} | 1 \rangle\! \sum\limits_{i = 1}^{N}\!\! \left[\!\left(\!\langle f_{i} \rangle - \langle b \rangle\!\right) \langle {g_{i}^{m}} \rangle - \sum\limits_{l \neq m} \langle s_{l} \rangle \langle e_{l} | 1 \rangle \langle {g_{i}^{l}} {g_{i}^{m}} \rangle\! \right]. \end{aligned}} $$

We define the factors for the third part of our probabilistic model as

$$\begin{aligned} q(\gamma) &= \text{Gamma}(\gamma; \alpha(\gamma), \beta(\gamma)) \\ q(b) &= \text{Normal}(b; \mu(b), \Sigma(b)) \\ q(\boldsymbol{f}) &= \prod \limits_{i = 1}^{N} \text{TruncatedNormal}(f_{i}; \mu(f_{i}), \Sigma(f_{i}), \rho(f_{i})), \end{aligned} $$

where TruncatedNormal(·;***μ***,**Σ**,*ρ*(·)) denotes the truncated normal distribution with the mean vector ***μ***, the covariance matrix **Σ** and the truncation rule *ρ*(·) such that TruncatedNormal(·;***μ***,**Σ**,*ρ*(·))∝Normal(·;***μ***,**Σ**) if *ρ*(·) is true, and TruncatedNormal(·;***μ***,**Σ**,*ρ*(·))=0 otherwise. The approximate posterior distributions can be updated as

$$\begin{aligned} \alpha(\gamma) &= \alpha_{\gamma} + 1/2 \\ \beta(\gamma) &= (1/\beta_{\gamma} + \langle b^{2} \rangle/2)^{-1} \\ \Sigma(b) &= (\langle \gamma \rangle + N)^{-1} \\ \mu(b) &= \Sigma(b) \left(\sum \limits_{i = 1}^{N} \langle f_{i} \rangle - \langle (\boldsymbol{s} \circ \boldsymbol{e})^{\top} \rangle \langle \boldsymbol{g}_{i} \rangle\right) \\ \Sigma(f_{i}) &= 1 \\ \mu(f_{i}) &= \langle (\boldsymbol{s} \circ \boldsymbol{e})^{\top} \rangle \langle \boldsymbol{g}_{i} \rangle + \langle b \rangle \\ \rho(f_{i}) &\triangleq f_{i} y_{i} > \nu, \end{aligned} $$

where we can fortunately calculate the expectation of the truncated normal distribution in closed-form.

#### Prediction scenario

We can replace $p\left (\boldsymbol {a} | \{\mathbf {K}_{m}\}_{m = 1}^{P}, \boldsymbol {y}\right)$ with its approximate posterior distribution *q*(***a***) and obtain the posterior predictive mean of the latent variables ***g***_⋆_ for a new data point ***x***_⋆_ as

$$\begin{aligned} \langle \boldsymbol{g}_{\star} \rangle &= [\boldsymbol{k}_{1,\star} ~ \hdots ~ \boldsymbol{k}_{P,\star}]^{\top} \langle \boldsymbol{a} \rangle. \end{aligned} $$

The posterior predictive mean of the predicted output *f*_⋆_ can also be found by replacing $p(b, \boldsymbol {e}, \boldsymbol {s} | \{\mathbf {K}_{m}\}_{m = 1}^{P}, \boldsymbol {y})$ with its approximate posterior distribution *q*(*b*)*q*(***e***|***s***)*q*(***s***):

$$\begin{aligned} \langle f_{\star} \rangle &= \langle (\boldsymbol{s} \circ \boldsymbol{e})^{\top} \rangle \langle \boldsymbol{g}_{\star} \rangle + \langle b \rangle, \end{aligned} $$

where we use 〈*f*_⋆_〉 to predict the class label by looking at its sign.

### Sparse Bayesian multitask multiple kernel learning

We formulate the joint modeling of prediction tasks on multiple datasets using a multitask learning approach, which models distinct but related tasks conjointly to improve overall generalization performance. We are given *T* datasets, and, for each dataset, we have an independent and identically distributed sample $\{\boldsymbol {x}_{t,i} \in \mathcal {X}\}_{i = 1}^{N_{t}}$ and a class label vector $\boldsymbol {y}_{t} = \{y_{t,i} \in \mathcal {Y}\}_{i = 1}^{N_{t}}$, where *N*_*t*_ is the number of data points in the dataset *t*. We also have a list of gene sets $\{\mathcal {I}_{m}\}_{m = 1}^{P}$, which are shared across the tasks, and the corresponding kernel functions $\{k_{t, m}(\cdot, \cdot | \mathcal {I}_{m})\}_{m = 1}^{P}$ for each task.

#### Probabilistic model

Our single-task learning model SBMKL is extended towards multitask learning to obtain ‘sparse Bayesian multitask multiple kernel learning’ (SBMTMKL).

The distributional assumptions of the first part can be modified as

$${{}\begin{aligned} \lambda_{t,i} &\sim \text{Gamma}(\lambda_{t,i}; \alpha_{\lambda}, \beta_{\lambda}) &&\;\;\;\;\;\; \forall (t, i) \\ a_{t,i} | \lambda_{i} &\sim \text{Normal}\left(a_{t,i}; 0, \lambda_{t,i}^{-1}\right) &&\;\;\;\;\;\; \forall (t, i) \\ g_{t,i}^{m} | \boldsymbol{a}_{t}, \boldsymbol{k}_{t,m,i} &\sim \text{Normal}\left(g_{t,i}^{m}; \boldsymbol{a}_{t}^{\top} \boldsymbol{k}_{t,m,i}, {\sigma_{g}^{2}}\right) &&\;\;\;\;\;\; \forall (t, m, i), \end{aligned}} $$

where we have task-specific model variables and latent variables.

The distributional assumptions of the second part are written as

$$\begin{aligned} \kappa &\sim \text{Beta}(\kappa; \zeta_{\kappa}, \eta_{\kappa}) && \\ s_{m} | \kappa &\sim \text{Bernoulli}(s_{m}; \kappa) &&\;\;\;\;\;\; \forall m \\ \omega_{t} &\sim \text{Gamma}(\omega_{t}; \alpha_{\omega}, \beta_{\omega}) &&\;\;\;\;\;\; \forall t \\ e_{t,m} | \omega_{t} &\sim \text{Normal}(e_{t, m}; 0, \omega_{t}^{-1}) &&\;\;\;\;\;\; \forall (t, m), \end{aligned} $$

where the binary indicator variables are shared across the tasks, which helps us transfer information between them.

The distributional assumptions of the third part can be modified as

$${{\begin{aligned} \gamma_{t} &\sim \text{Gamma}\left(\gamma_{t}; \alpha_{\gamma}, \beta_{\gamma}\right) &&\;\;\;\;\;\; \forall t \\ b_{t} | \gamma_{t} &\sim \text{Normal}\left(b_{t}; 0, \gamma_{t}^{-1}\right) &&\;\;\;\;\;\; \forall t \\ f_{t,i} | b_{t}, \boldsymbol{e}_{t}, \boldsymbol{s}, \boldsymbol{g}_{t,i} &\sim \text{Normal}\left(f_{t,i}; (\boldsymbol{s} \circ \boldsymbol{e}_{t})^{\top} \boldsymbol{g}_{t,i} + b_{t}, 1\right) &&\;\;\;\;\;\; \forall (t, i) \\ y_{t,i} | f_{t,i} &\sim \text{Kronecker}\left(f_{t,i} y_{t,i} > \nu\right) &&\;\;\;\;\;\; \forall (t, i), \end{aligned}}} $$

where we have task-specific bias parameters and predicted outputs.

#### Inference using variational Bayes

We approximate the posterior distribution over the model parameters and the latent variables by a variational distribution:

$$\begin{aligned} p\left(\mathbf{\Theta} | \{\{\mathbf{K}_{t, m}\}_{m = 1}^{P}, \boldsymbol{y}_{t}\}_{t = 1}^{T}\right) &\approx q(\mathbf{\Theta}), \end{aligned} $$

where we start inference by writing *q*(**Θ**) as a factorized approximation:

$${{}{\begin{aligned} q(\mathbf{\Theta}) &= \prod\limits_{t = 1}^{T} \Bigl[q(\boldsymbol{\lambda}_{t}) q(\boldsymbol{a}_{t}) q(\mathbf{G}_{t})\Bigr] q(\kappa) q(\boldsymbol{s}) \prod\limits_{t = 1}^{T} \Bigl[q(\omega_{t}) q(\boldsymbol{e}_{t} | \boldsymbol{s})\Bigr]\\ &\quad\times \prod\limits_{t = 1}^{T} \Bigl[q(\gamma_{t}) q(b_{t}) q(\boldsymbol{f}_{t})\Bigr]. \end{aligned}}} $$

#### Inference details

The update equations of the approximate posterior distributions for all model parameters and latent variables are very similar to those of SBMKL except for the binary indicator variables. We can update the approximate posterior distribution of them as

$$\begin{aligned} \pi(s_{m}) &= 1 / (1 + \exp(-r_{m})) \end{aligned} $$

where the auxiliary variable *r*_*m*_ is defined as

$${{\begin{aligned} r_{m} &= \left\langle\log\frac{\kappa}{1 - \kappa}\right\rangle - \frac{1}{2} \sum\limits_{t = 1}^{T} \langle e_{t,m}^{2} | 1 \rangle \langle \boldsymbol{g}_{t}^{m} (\boldsymbol{g}_{t}^{m})^{\top} \rangle \\ & \quad + \! \sum\limits_{t = 1}^{T} \langle e_{t, m} | 1 \rangle \sum\limits_{i = 1}^{N_{t}} \left[\left(\langle f_{t,i} \rangle \,-\, \langle b_{t} \rangle\right) \langle g_{t,i}^{m} \rangle \,-\, \sum\limits_{l \neq m} \langle s_{l} \rangle \langle e_{t,l} | 1 \rangle \langle g_{t,i}^{l} g_{t,i}^{m} \rangle \! \right]. \end{aligned}}} $$

#### Prediction scenario

We can use the approximate posterior distribution *q*(***a***_*t*_) instead of $p(\boldsymbol {a}_{t}| \{\{\mathbf {K}_{t, m}\}_{m = 1}^{P}, \boldsymbol {y}_{t}\}_{t = 1}^{T})$ and obtain the posterior predictive mean of the latent variables ***g***_*t*,⋆_ for a new data point ***x***_*t*,⋆_ in the task *t* as

$$\begin{aligned} \langle \boldsymbol{g}_{t,\star} \rangle &= [\boldsymbol{k}_{t,1,\star} ~ \hdots ~ \boldsymbol{k}_{t,P,\star}]^{\top} \langle \boldsymbol{a}_{t} \rangle. \end{aligned} $$

The posterior predictive mean of the predicted output *f*_*t*,⋆_ can also be found by replacing $p\left (b_{t}, \boldsymbol {e}_{t}, \boldsymbol {s} | \left \{ \{\mathbf {K}_{t, m}\}_{m = 1}^{P}, \boldsymbol {y}_{t}\right \}_{t = 1}^{T}\right)$ with its approximate posterior distribution *q*(*b*_*t*_)*q*(***e***_*t*_|***s***)*q*(***s***):

$$\begin{aligned} \langle f_{t,\star} \rangle &= \langle (\boldsymbol{s} \circ \boldsymbol{e}_{t})^{\top} \rangle \langle \boldsymbol{g}_{t,\star} \rangle + \langle b_{t} \rangle, \end{aligned} $$

where we use 〈*f*_*t*,⋆_〉 to predict the class label by looking at its sign.

### Baseline algorithm

We use a kernelized Bayesian classification algorithm, which is known as relevance vector machine [24], as the baseline algorithm. Its distributional assumptions are defined as

$$\begin{aligned} \lambda_{i} &\sim \text{Gamma}\left(\lambda_{i}; \alpha_{\lambda}, \beta_{\lambda}\right) &&\;\;\;\;\;\; \forall i \\ a_{i} | \lambda_{i} &\sim \text{Normal}\left(a_{i}; 0, \lambda_{i}^{-1}\right) &&\;\;\;\;\;\; \forall i \\ \gamma &\sim \text{Gamma}\left(\gamma; \alpha_{\gamma}, \beta_{\gamma}\right) && \\ b | \gamma &\sim \text{Normal}\left(b; 0, \gamma^{-1}\right) && \\ f_{i} | \boldsymbol{a}, b, \boldsymbol{k}_{i} &\sim \text{Normal}\left(f_{i}; \boldsymbol{a}^{\top} \boldsymbol{k}_{i} + b, 1\right) &&\;\;\;\;\;\; \forall i \\ y_{i} | f_{i} &\sim \text{Kronecker}\left(f_{i} y_{i} > \nu\right) &&\;\;\;\;\;\; \forall i, \end{aligned} $$

where the predicted outputs of data points are modeled as a linear function of their kernel representations (i.e. ***a*** ^⊤^ ***k***_*i*_+*b*). We again learn the posterior distribution over the model parameters and the latent variables using a deterministic variational approximation as we do for our methods. We call this algorithm ‘Bayesian relevance vector machine’ (BRVM). We have three main reasons for choosing this particular baseline algorithm: (i) BRVM can make use of kernel functions to obtain nonlinear models like our methods. (ii) We can see the effect of using gene set information by comparing our methods to BRVM. (iii) BRVM uses the same type of inference mechanism with our methods.

## Results and discussion

To illustrate the effectiveness of our proposed methods SBMKL and SBMTMKL, we report their results on four datasets (i.e. two cancer and two tuberculosis datasets) and compare them to the baseline algorithm BRVM, which does not make use of gene set information, using repeated random subsampling validation experiments.

### Experimental settings

For each dataset, we create 100 random train/test splits to obtain robust results. For each replication, the training set is defined by randomly selecting 75 % of the data points with stratification on the phenotype, and the remaining 25 % of the samples are used as the test set. The training set is normalized to have zero mean and unit standard deviation, and the test set is then normalized using the mean and the standard deviation of the original training set.

We extract gene sets from ‘the Molecular Signatures Database’ (MSigDB) [3], which contains curated pathway gene sets from online databases such as ‘the Kyoto Encyclopedia of Genes and Genomes’ (KEGG) [25] and ‘the Pathway Interaction Database’ (PID) [26]. In our experiments, we use 196 PID pathways reported in MSigDB as our gene set collection.

To calculate similarity between data points for all methods, we use the Gaussian kernel:

$$\begin{aligned} k_{\text{Gaussian}}(\boldsymbol{x}_{i}, \boldsymbol{x}_{j}) &= \exp \left(-\|\boldsymbol{x}_{i} - \boldsymbol{x}_{j}\|_{2}^{2} / (2 s^{2})\right), \end{aligned} $$

where ∥·∥_2_ denotes the *ℓ*_2_-norm, and we set the kernel width *s* to the mean of pairwise Euclidean distances between the data points:

$$\begin{aligned} s &= \frac{1}{N^{2}} \sum\limits_{i = 1}^{N} \sum\limits_{j = 1}^{N} \|\boldsymbol{x}_{i} - \boldsymbol{x}_{j}\|_{2}. \end{aligned} $$

For BRVM, we calculate a single kernel over all input features. For SBMKL and SBMTMKL, we calculate a separate kernel function for each gene set over the corresponding features. Note that the Gaussian kernels calculated on the gene sets take values between 0 and 1 by definition, and there is no need for eliminating small/large gene sets or performing additional normalization steps to remove the effect of gene set size.

The hyper-parameter values of BRVM are selected as (*α*_*λ*_,*β*_*λ*_)=(1,1), (*α*_*γ*_,*β*_*γ*_)=(1,1) and *ν*=1. The hyper-parameter values of SBMKL and SBMTMKL are selected as (*α*_*λ*_,*β*_*λ*_)=(1,1),*σ*_*g*_=0.1,(*ζ*_*κ*_,*η*_*κ*_)=(1,999),(*α*_*ω*_,*β*_*ω*_)=(1,1),(*α*_*γ*_,*β*_*γ*_)=(1,1) and *ν*=1. Note that (*ζ*_*κ*_,*η*_*κ*_) are set to these particular values to produce very sparse binary indicator variables, leading to classifiers with very few gene sets used for prediction. For BRVM, we perform 200 iterations during variational inference, whereas we perform 50 iterations for SBMKL and SBMTMKL.

We use ‘area under the receiver operating characteristic curve’ (AUROC) to compare classification results. AUROC is used to summarize the receiver operating characteristic curve, which is a curve of true positives as a function of false positives while the threshold to predict labels changes. Larger AUROC values correspond to better performance.

### Classification results on the cancer datasets

**Fig. 3:**
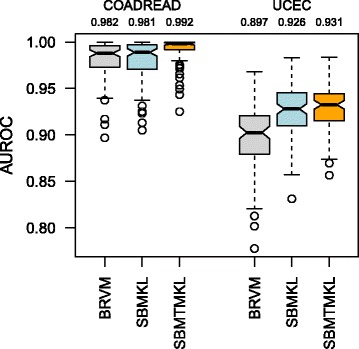
AUROC values on the cancer datasets for MSI-H versus others classification. The *box-and-whisker plot* shows the results over 100 replications in repeated random subsampling validation experiments of BRVM, SBMKL and SBMTMKL on both datasets. The numbers just below the dataset names give the average AUROC value for each experiment

On the cancer datasets, we run binary classification experiments to separate MSI-H patients from others (i.e. MSI-L and MSS), which is in agreement with the earlier studies that combine MSI-L and MSS tumors into the same group [11]. For BRVM and SBMKL methods, we train a separate classification model on each dataset, whereas, for SBMTMKL, we train a joint model on both datasets. Figure 3 compares the performance of BRVM, SBMKL and SBMTMKL on both datasets in terms of AUROC over 100 replications using box-and-whisker plots, and also reports the average AUROC value for each experiment. We clearly see that our methods with sparse gene set weights, leading to classifiers with very few active features, obtain results comparable to or even better than BRVM. Note that BRVM uses all available input features of the genomic data for classification. For example, SBMKL falls behind BRVM just by 0.1 % on COADREAD dataset, but obtains 2.9 % higher average AUROC on UCEC dataset. The average AUROC values become even higher if we model both datasets together using our multitask learning method SBMTMKL, which outperforms BRVM by 1.0 and 3.4 % on COADREAD and UCEC, respectively. Our sparse classifiers obtain these results using very few active features (i.e. features related to the genes in the gene sets with nonzero binary indicator variables); SBMKL uses 154.19 (3.40) and 403.03 (8.27) out of 20 530 (196) input features (gene sets) on the average, whereas SBMTMKL uses 484.03 (9.96) features (gene sets) on the average (i.e. less than 2.5 % of the input features) and obtains better classification results than BRVM and SBMKL on both datasets.

### Classification results on the tuberculosis datasets

**Fig. 4:**
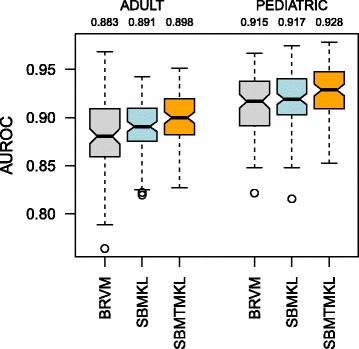
AUROC values on the tuberculosis datasets for ATB versus others classification. The *box-and-whisker plots* show the results over 100 replications in repeated random subsampling validation experiments of BRVM, SBMKL and SBMTMKL on both datasets. The numbers just below the dataset names give the average AUROC value for each experiment

On the tuberculosis datasets, we perform binary classification experiments to separate individuals with ATB from others (i.e. individuals with LTBI or OD), which is critical in clinical settings because we should correctly identify individuals who need tuberculosis treatment [14]. Figure 4 compares the performance of BRVM, SBMKL and SBMTMKL on both datasets. We see that our methods obtain results better than BRVM. On ADULT and PEDIATRIC datasets, SBMKL outperforms BRVM by 0.8 and 0.2 % using 782.21 (11.41) and 569.51 (7.88) out of 47, 323 (196) input features (gene sets) on the average, respectively. Our multitask learning method SBMTMKL again has the highest AUROC values on both datasets and outperforms BRVM by 1.5 % on ADULT and 1.3 % on PEDIATRIC using 1 102.65 (16.07) features (gene sets) on the average.

### Biological results on the cancer datasets

To illustrate the biological relevance of our methods, we analyze their abilities to identify relevant gene sets based on the binary indicator variables inferred during training. For each gene set, we count the number of replications in which the corresponding binary indicator variable is nonzero. Table 3 lists the top 10 most frequently selected gene sets together with their selection frequencies for three scenarios: (i) SBMKL on COADREAD, (ii) SBMKL on UCEC and (iii) SBMTMKL on COADREAD and UCEC. We see that SBMKL is able to identify WNT_NONCANONICAL_PATHWAY and TGFBRPATHWAY as the top two gene sets in the first scenario, which are reported to be involved in the initiation and progression of colorectal cancer [12]. However, their selection frequencies are quite low (i.e. less than or equal to 0.10). Similarly, for UCEC, it is able to identify two apoptosis-related gene sets, namely, P53DOWNSTREAMPATHWAY and NOTCH_PATHWAY, as the top gene sets with more than 0.80 frequencies, which are known to be associated with endometrial cancer [13]. When we jointly model both datasets using our multitask learning method SBMTMKL, we are able to identify P53DOWNSTREAMPATHWAY, NOTCH_PATHWAY and WNT_NONCANONICAL_PATHWAY as the top gene sets with increased frequencies compared to those of SBMKL. We see that multitask learning decreases the effect of random subsampling by picking relevant gene sets more frequently, leading to more robust knowledge extraction for both datasets.

**Table 3:** Gene set selection results on the cancer datasets for MSI-H versus others classification

SBMKL on COADREAD		SBMKL on UCEC		SBMTMKL on COADREAD and UCEC	
Gene set name	Frequency	Gene set name	Frequency	Gene set name	Frequency
WNT_NONCANONICAL_PATHWAY	0.10	P53DOWNSTREAMPATHWAY	0.92	P53DOWNSTREAMPATHWAY	0.99
TGFBRPATHWAY	0.09	NOTCH_PATHWAY	0.83	NOTCH_PATHWAY	0.92
DELTANP63PATHWAY	0.07	NFAT_TFPATHWAY	0.26	WNT_NONCANONICAL_PATHWAY	0.61
TAP63PATHWAY	0.07	IL5_PATHWAY	0.24	NFAT_TFPATHWAY	0.41
RB_1PATHWAY	0.07	P53REGULATIONPATHWAY	0.24	AR_PATHWAY	0.34
NFAT_3PATHWAY	0.06	CDC42_REG_PATHWAY	0.20	RHOA_PATHWAY	0.21
ATF2_PATHWAY	0.06	AVB3_OPN_PATHWAY	0.15	REG_GR_PATHWAY	0.21
SMAD2_3NUCLEARPATHWAY	0.05	WNT_NONCANONICAL_PATHWAY	0.14	UPA_UPAR_PATHWAY	0.17
P73PATHWAY	0.05	REG_GR_PATHWAY	0.13	BMPPATHWAY	0.17
MYC_ACTIVPATHWAY	0.05	UPA_UPAR_PATHWAY	0.11	RAC1_PATHWAY	0.14

The table displays the top 10 most frequently selected gene sets together with their selection frequencies for three scenarios

**Fig. 5:**
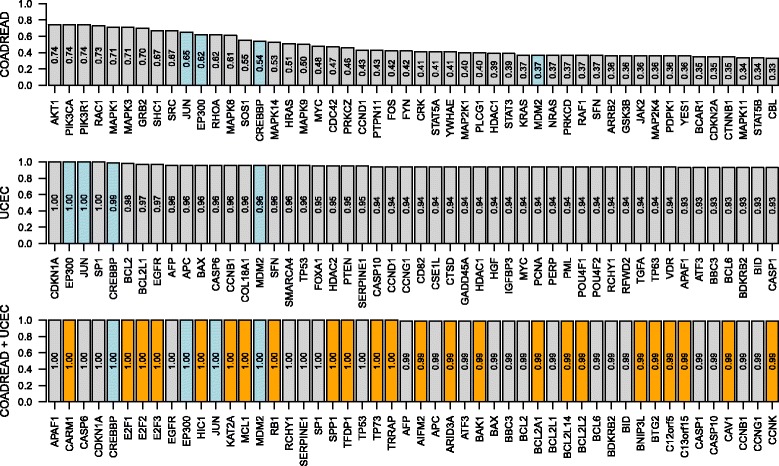
Gene selection results on the cancer datasets for MSI-H versus others classification. The *bar plots* display the top 50 most frequently selected genes together with their selection frequencies for three scenarios. *Blue bars* show the genes that are in the top 50 for all scenarios, and *orange bars* show the genes that are in the top 50 only for multitask learning scenario

We also count the number of replications for each gene in which it is included in the final classifier. Figure 5 displays the top 50 most frequently selected genes together with their selection frequencies for three scenarios. CREBBP, EP300, JUN and MDM2 are among the top 50 genes for all scenarios, which is reasonable considering their functions in cell cycle. We see that the selection frequencies of the first two scenarios are lower than those of the third scenario, which is consistent with our gene set selection results. Our multitask learning method SBMTMKL includes several genes in the top 50 that are not selected by SBMKL in two other scenarios, which may lead to interesting findings. For example, E2F1, E2F2 and E2F3 are used in the final classifier in all replications, which are reported to be related to cellular proliferation [27].

### Biological results on the tuberculosis datasets

We also evaluate the gene set selection results of our methods on the tuberculosis datasets. Table 4 lists the top 10 most frequently selected gene sets together with their selection frequencies for three scenarios: (i) SBMKL on ADULT, (ii) SBMKL on PEDIATRIC and (iii) SBMTMKL on ADULT and PEDIATRIC. We see that the gene set selection frequencies of SBMKL on PEDIATRIC dataset are quite low (i.e. between 0.13 and 0.31) compared to those on ADULT dataset. However, when we model both datasets using our multitask learning method SBMTMKL, the selection frequencies of the top 10 gene sets become significantly higher (i.e. between 0.35 and 0.67), leading to more robust gene set signatures.

**Table 4:** Gene set selection results on the tuberculosis datasets for ATB versus others classification

SBMKL on ADULT		SBMKL on PEDIATRIC		SBMTMKL on ADULT and PEDIATRIC	
Pathway name	Frequency	Pathway name	Frequency	Pathway name	Frequency
ERBB_NETWORK_PATHWAY	0.73	A6B1_A6B4_INTEGRIN_PATHWAY	0.31	RHODOPSIN_PATHWAY	0.67
AP1_PATHWAY	0.55	RAS_PATHWAY	0.27	ERBB_NETWORK_PATHWAY	0.63
CONE_PATHWAY	0.44	INTEGRIN1_PATHWAY	0.24	AP1_PATHWAY	0.60
AR_TF_PATHWAY	0.42	RAC1_PATHWAY	0.21	SYNDECAN_1_PATHWAY	0.51
CERAMIDE_PATHWAY	0.31	RHODOPSIN_PATHWAY	0.20	PLK1_PATHWAY	0.50
RHODOPSIN_PATHWAY	0.31	SYNDECAN_1_PATHWAY	0.17	CERAMIDE_PATHWAY	0.42
SYNDECAN_1_PATHWAY	0.29	ATM_PATHWAY	0.16	ATM_PATHWAY	0.41
FANCONI_PATHWAY	0.25	ATF2_PATHWAY	0.15	AR_TF_PATHWAY	0.40
RXR_VDR_PATHWAY	0.24	THROMBIN_PAR1_PATHWAY	0.15	ATF2_PATHWAY	0.37
HNF3BPATHWAY	0.23	IL12_2PATHWAY	0.13	HNF3APATHWAY	0.35

The table displays the top 10 most frequently selected gene sets together with their selection frequencies for three scenarios

**Fig. 6:**
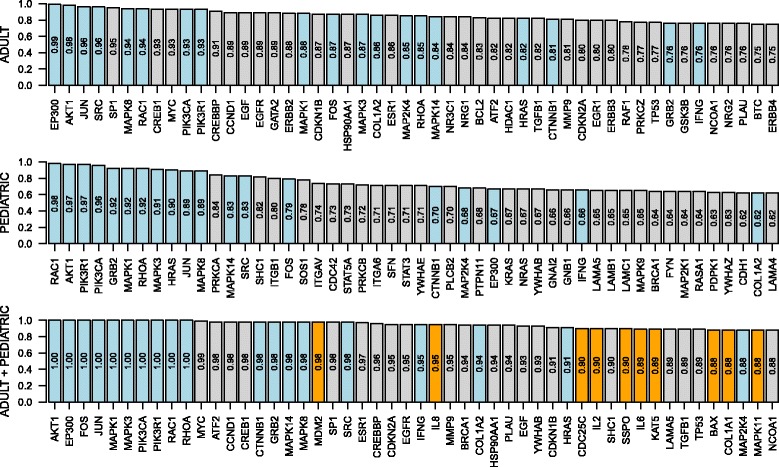
Gene selection results on the tuberculosis datasets for ATB versus others classification. The *bar plots* display the top 50 most frequently selected genes together with their selection frequencies for three scenarios. *Blue bars* show the genes that are in the top 50 for all scenarios, and *orange bars* show the genes that are in the top 50 only for multitask learning scenario

Figure 6 displays the top 50 most frequently selected genes together with their selection frequencies for three scenarios. We see that the genes that are part of signaling mechanisms such as MAPK1, MAPK3, MAPK8, PIK3CA, PIK3R1 and RAC1 are selected in the top 50 genes for all scenarios. Similar to the results on the cancer datasets, the selection frequencies of the first two scenarios are lower than those of the third scenario, which shows the robustness of multitask learning approach. As an interesting finding, SBMTMKL includes three genes from interleukin family, namely, IL8, IL2 and IL6, in the top 50, which are shown to be diagnostically associated with tuberculosis [28–30], whereas they are not picked in the top 50 by SBMKL in single dataset experiments.

## Conclusions

Integrating gene set analysis and predictive modeling is already considered by many existing studies, which fail either to capture nonlinear dependencies between genomic data and phenotype or to model multiple related datasets conjointly.

In this study, we integrate gene set analysis and nonlinear predictive modeling of disease phenotypes by casting this problem into a binary classification framework defined on the gene sets with a sparsity-inducing prior on their weights. To this aim, we propose a Bayesian multiple kernel learning algorithm, which produces a classifier with sparse gene set weights, by extending our earlier Bayesian formulation [8]. We then generalize this new algorithm to multitask learning to be able to model multiple related datasets conjointly, leading to better generalization performance and to more robust molecular signatures. The main novelty of our methods is the integration of gene set analysis and nonlinear predictive modeling using a probabilistic formulation, which enables us to robustly capture nonlinear dependencies between genomic data and phenotype even with small sample sizes, and to use overlapping gene sets and gene sets with different sizes without any major concern. Our approach brings us two side advantages: (i) We can identify very few gene sets predictive of the phenotype, which may shed light on underlying biological processes. (ii) We can reduce the data acquisition cost for test samples in clinical settings by collecting only the features used in our classifier.

To demonstrate the performance of our algorithms SBMKL and SBMTMKL, we perform repeated random subsampling validation experiments on four datasets of two major human diseases, namely, cancer and tuberculosis. On the two cancer datasets [12, 13], we decide whether a colorectal or endometrial tumor displays micro-satellite instability using its mRNA gene expression data. On the two tuberculosis datasets [14, 15], we diagnose whether an adult or pediatric individual has an active tuberculosis infection using his/her whole blood RNA expression data. We compare our two methods to a baseline Bayesian nonlinear algorithm that is trained on all available genomic data without using gene set information. Our methods obtain comparable or even better predictive performance using very few features (i.e. less than 2.5 % of the input features) on all datasets. We also show that we are able to identify biologically relevant genes and gene sets for cancer and tuberculosis phenotypes, which are validated by the existing studies from the literature. The results of our multitask learning algorithm show that modeling multiple related datasets conjointly enables us to further improve the generalization performance and to better understand biological processes behind disease phenotypes.

In the experiments reported, we use real-valued gene expression measurements as genomic data. Our methods can also be applied to discrete data such as mutation profiles of tumors, which are hard to use in classical gene set analysis methods due to their very sparse nature. As a possible extension, we plan to use our kernel-based formulations on cancer datasets to identify driver mutations using kernels for discrete data such as the Jaccard similarity coefficient.
